# How to Help the Suicidal Person to Choose Life: The Ethic of Care and Empathy as an Indispensable Tool for Intervention by K. Stephany

**DOI:** 10.2174/1874434601711010262

**Published:** 2017-12-29

**Authors:** John R. Cutcliffe

**Affiliations:** 1 Owner/Operator: Cutcliffe Consulting (Operating in Canada, USA and Europe), Amherstview, Ontario; 2 Adjunct Professor: School of Nursing, University of Coimbra, Portugal; 3 Associate Editor: International Journal of Mental Health Nursing

In comparison with four major contemporary causes of death in the Occidental world, the progress we have made in combating rising rates of suicide is not encouraging. During recent decades, the significant decreases in death rates from cancer, heart disease, tuberculosis and cerebral vascular accidents (*e.g*. strokes) have not been seen in suicide/suicide prevention. Regretfully, the rate of suicide in the general population during recent decades has trended upwards. In the light of these data, those involved in the care of the suicidal person might welcome additional literature that has the potential to help address the alarming rate of suicide and thus I welcome the publication of this new book. 

Some of the central issues included in this book, while not necessarily new or original, are nevertheless worthy of scrutiny:

1. The clear and well documented need for greatly improved training and education in the care of the suicidal person,
2. The centrality of empathy to working therapeutically with the suicidal person,
3. And listening to and subsequently inducing theory from people who have personally experienced care to help address their increased suicide risk, Are important and this book offers some interesting perspectives on these issues? Readers interested in learning more about how to work with suicidal people then might benefit from reviewing the material included in this book. I certainly encourage readers to do so.

At times, as one reads through the book, one gets the sense that the author has perhaps attempted to include and cover too many topics or areas, resulting in a text that, at times, lacks parsimony. Moreover, the design and layout of the text, in places, look and feel a bit disjointed and jumbled.

This is for those who are interested in care of the suicidal person, there is ample material available in this book that is worth to read.

